# Identification of a novel GLA mutation (F69 L) in a Japanese patient with late-onset Fabry disease

**DOI:** 10.1038/hgv.2015.44

**Published:** 2015-11-12

**Authors:** Toshiko Umeda, Seiji Hashimoto, Kazuyuki Noriyasu, Ayumi Takamura, Miwa Fujisaki, Yoshikatsu Eto

**Affiliations:** 1 Advanced Clinical Research Center, Institute for Neurological Disorders, Kanagawa, Japan; 2 Department of Nephrology, NTT East Japan Sapporo Hospital, Sapporo, Japan; 3 Department of Cardiovascular Medicine, NTT East Japan Sapporo Hospital, Sapporo, Japan; 4 Department of Biological Regulation School of Health Science Faculty of Medicine, Tottori University, Tottori, Japan; 5 Jikei University School of Medicine, Tokyo, Japan

## Abstract

Fabry disease is an X-linked recessive inborn error of glycosphingolipid catabolism caused by a mutation in the *GLA* gene. We sequenced the α-galactosidase A gene (*GLA*) of a patient who had been clinically diagnosed with late-onset Fabry disease. Abundant globotriaosylceramide was present in his urine, which indicated typical Fabry disease. Here, we report a novel hemizygous mutation, c.207C>A (Phe69 Leu), which caused a mild/late-onset form of Fabry disease.

Fabry disease (OMIM #301500) is an X-linked lysosomal storage disease that is characterized by a deficiency in the production of lysosomal exoglycohydrolase α-galactosidase A (α-d-galactoside galactohydrolase, EC 3.2.1.22; αGal-A).^1,2^ Absent or deficient αGal-A activity results in progressive accumulation of globotriaosylceramide (Gb_3_) and related glycosphingolipids (Gb_2_, Lyso-Gb_3_) within lysosomes in a variety of cell types, including capillary endothelial cells, renal cells (podocytes and tubular, glomerular endothelial, mesangial and interstitial cells), cardiac cells (cardiomyocytes and fibroblasts) and nerve cells.^3,4^ This accumulation impairs cellular functions. Patients with advanced Fabry disease present with renal function deterioration, hypertrophic cardiomyopathy and cerebrovascular involvement and are increasingly at risk of developing complications, including end-stage renal failure, stroke, cardiac fibrosis, congestive heart failure, and finally, death.^5^ The clinical spectrum of Fabry disease ranges from the classic form, which appears in early childhood, to the late-onset form, which is diagnosed in adulthood. Classic Fabry disease patients exhibit the following symptoms: angiokeratomas, pain in the extremities, reduced or absent sweating, gastrointestinal upset, and characteristic corneal opacities. With advancing age, clinical manifestations include renal insufficiency, cardiac involvement and cerebrovascular disease. Conversely, late-onset Fabry disease has renal or cardiac involvement without characteristic skin lesions or pain crises and is usually diagnosed when the patient is in his 50 s or 60 s. Fabry disease is usually diagnosed by measurement of αGal-A activity in plasma, leukocytes or dried whole blood spots (DBS) on filter paper.^6,7^ Gb3 and Lyso-Gb3 levels in plasma or urine can aid in the diagnosis of Fabry disease.^8,9^

The 14-kb α-galactosidase A gene (*GLA*) is located at Xq22.1 and consists of seven exons (GenBank accession X14448). The 1.45-kb *GLA* mRNA (GenBank accession NM_000169) encodes a polypeptide of 429 amino acids, including a 31 amino-acid N-terminal signal peptide. More than 650 mutations in *GLA* have previously been identified in Fabry disease patients (HGMD, http://www.hgmd.cf.ac.uk/ac/index.php). The estimated disease incidence ranges from 1 in 1250 to 1 in 11700 live male births.^10–13^

Here, we report a new *GLA* mutation in a male Fabry disease patient who was born in Hokkaido, Japan. His mother lived to be over 90-year old with a pacemaker, which is longer than the average life span of Japanese women. He did not have any symptoms, and thus, had not undergone a physical examination to obtain a Fabry disease diagnosis until he was 50-year old, at which time echocardiography demonstrated high-voltage potential and inverted T waves at I, aVL and V4-6. Echocardiography also demonstrated upper trabeculation. His interventricular septum thickness and left ventricular posterior wall thickness were 10.9 and 9.4 mm, respectively, which do not meet the definition of left ventricular hypertrophy. At 54 years of age, his physical examination revealed proteinuria (1.28 g gCr^−1^) and fatty casts with inclusion bodies in his urinary sediment. When he was 57-year old, his echocardiography demonstrated even higher voltage potential and inverted T waves compared with when he was 50-year old (Figure 1), and echocardiography demonstrated that his interventricular septum thickness and left ventricular posterior wall thickness had increased to 21.8 and 20.0 mm, respectively, demonstrating marked left ventricular hypertrophy. He did not display any cardiac arrhythmias, such as ventricular tachycardia, but mild bradycardia was observed using a Holter monitor. These findings suggested a clinical diagnosis of late-onset Fabry disease, although the patient did not show some Fabry disease features such as cornea verticillata, hypo-anhidrosis, skin lesions and pain crises. We then performed the following analyses: measurement of αGal-A activity in DBS using a fluorometric enzyme assay,^7^ measurement of Gb3 accumulation in urine using thin-layer chromatography to separate the components of the urine and sulfuric-acid spray to detect Gb3,^8^ and measurement of Lyso-Gb3 level in plasma using high-performance liquid chromatography.^9^

The patient’s αGal-A activity in DBS was markedly lower than the mean value of the controls (1.56 pmol per punch h^−1^ versus 11.82±3.99 pmol per punch h^−1^); the cutoff value that indicates below normal αGal-A activity in DBS is 4.01 pmol per punch per h^−1^. The patient’s lyso-Gb3 plasma level was increased to 12 nmol l^−1^ compared with the control (below the detection level of 2 nmol l^−1^).^9^ Moreover, thin-layer chromatography demonstrated that the patient’s urinary Gb3 level was markedly increased over the normal level (data not shown). With these findings, the patient was diagnosed with Fabry disease. Next, we sequenced the *GLA* gene to try to identify a causative mutation for the Fabry phenotype.

After genetic counseling and obtaining written informed consent, we obtained genomic DNA from the peripheral blood using a standard technique. We assessed all of the *GLA* exons and the flanking intronic regions by direct sequencing of PCR products. We identified one hemizygous mutation in exon 2 of *GLA*, c.207C>A (p. Phe69 Leu). The mutation was absent from the dbSNP and Human Genetic Variation Database databases. Because the mother’s DNA was not available, this mutation could not be confirmed to be *de novo*.

We sought to determine whether the Phe69 Leu mutation in *GLA*-altered protein function contributed to Fabry disease using automated methods available on the Internet, including Sorting Intolerant from Tolerant amino-acid substitutions (SIFT) (http://provean.jcvi.org/seq_submit.php),^14^ Polymorphism phenotyping-2 (PolyPhen-2) (http://genetics.bwh.harvard.edu/pph2/),^15^ Grantham score difference (Align-GVGD) (http://agvgd.iarc.fr/agvgd_input.php),^16^ Mutation taster (http://www.mutationtaster.org/)^17^ and Mutation Assessor (http://mutationassessor.org/).^18,19^ These software programs likely generate different results depending on their algorithms. SIFT relies only on the evolutionary information regarding important amino acids that tend to be conserved across species, and the SIFT score ranges from 0 to 1. A mutation with a value between 0 and 0.05 is predicted to affect protein function. PolyPhen-2 predicts variants as benign, possibly damaging, or probably damaging based on both the evolutionary conservation of the amino-acid sequence and the structural properties of the protein. Its score ranges from 0.000 (most probably benign) to 1 (most probably damaging). Align-GVGD is based on evolutionary conservation and physiochemical information, such as polarity and molecular volume. It classifies eight ordered grades of mutations ranging from the most likely to be pathogenic to the least likely to be pathogenic. Mutation Assessor uses conservation patterns within aligned families (conservation score) and sub-families (specificity score of homologs) and thus attempts to account for functional shifts between protein sub-families. It predicts variants to be neutral or to have a low, medium, or high probability of affecting protein function. Mutation taster integrates information from different databases with the physico-chemical features of the affected residue to provide a fully concordant prediction of the resulting disease. Its score ranges from 0.0 (most probably benign) to 215 (most probably disease causing).

The SIFT, PolyPhen-2 and Mutation taster scores for the Phe69 Leu mutation in *GLA* were 0.01 (damaging), 0.991 (probably damaging) and 22 (predicted to be disease causing), respectively. Conversely, the Align-GVGD and Mutation Assessor scores for the Phe69 Leu mutation were Class C15 (one grade highly than Class C0) and low, respectively. The Phe69 Leu mutation is a substitution of a neutral, non-polar phenylalanine residue to a neutral, non-polar leucine residue. In addition, p.Phe69 is not an activity site (47, 92-93, 134, 168, 170, 203, 227 and 231 sites) or a substrate-binding site (sites 203–207 in *GLA*) (GenBank accession NP_000160).^20^ The predicted functional consequences of the Phe69 Leu mutation suggested that it caused abnormal αGal-A activity that is not marked but is sufficient to cause Fabry disease; this is in accordance with the fact that this patient displayed late-onset Fabry disease, which is milder than the classic form of the disease.

The patient has two daughters; one is 10 s and the other is 20 s. They have no Fabry symptoms, but they do have the Fabry obligate heterozygous Phe69 Leu mutation that we identified in their father. Their αGal-A activities in DBS were 3.65 pmol per punch h^−1^ and 4.50 pmol per punch h^−1^, respectively. The patient’s mother, who was likely a Fabry mutation carrier, lived a long time. She had a pacemaker implanted but did not have any other notable symptoms. Although female patients that are heterozygous for Fabry disease mutations show Fabry symptoms, they are usually milder than the symptoms of male Fabry disease patients.^1^ Patients who are heterozygous for the Phe69 Leu mutation may show few symptoms. We need to follow-up the female carriers of the Phe69 Leu mutation and more thoroughly review the clinical information of other family members of the patient in this case report.

In conclusion, we identified a novel missense mutation in *GLA*, Phe69 Leu, in a late-onset Fabry disease patient. The identification of this novel mutation will enable clinicians and genetic counselors to have a better clinical understanding of Fabry disease.

## Figures and Tables

**Figure 1:**
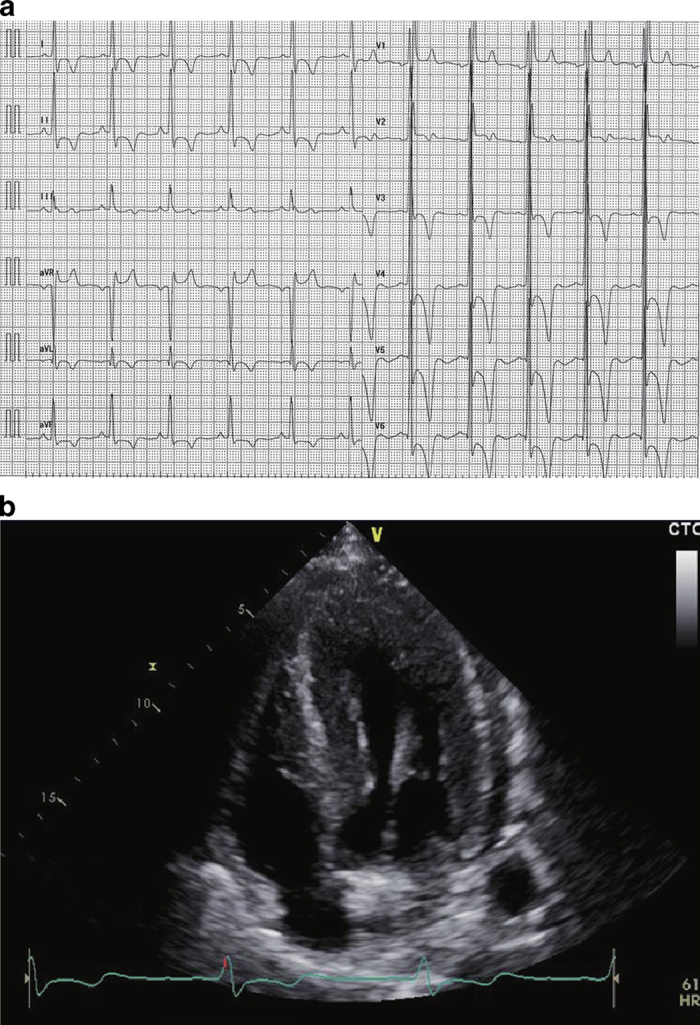
The patient’s ECG demonstrated marked left ventricular hypertrophy and an inverted T wave, which indicates ischemic changes in the cardiac muscle. The cardiac ECHO indicated ischemic changes and edema in the endocardium, as well as left ventricular hypertrophy, cardiac muscle calcification and valvular fibrosis.
